# Ultrasound-Guided Percutaneous Thermal Ablation of Hepatic Focal Nodular Hyperplasia——A Multicenter Retrospective Study

**DOI:** 10.3389/fbioe.2021.826926

**Published:** 2022-01-06

**Authors:** Xuan Yu, Jiandong Chang, Dezhi Zhang, Qiang Lu, Songsong Wu, Kai Li

**Affiliations:** ^1^ Department of Medical Ultrasonics, The Third Affiliated Hospital of Sun Yat-sen University, Guangdong, China; ^2^ Department of Ultrasound, Xiamen Chinese Medical Hospital, Fujian, China; ^3^ Department of Ultrasound, The First Hospital of Jilin University, Jilin, China; ^4^ Department of Ultrasound, West China Hospital, Sichuan, China; ^5^ Department of Ultrasonography, Shengli Clinical Medical College of Fujian Medical University, Fujian, China

**Keywords:** focal nodular hyperplasia, ultrasonography, radiofrequency ablation, microwave ablation, thermal ablation

## Abstract

**Background and Aim:** To evaluate the clinical effect of ultrasound (US)-guided percutaneous thermal ablation of hepatic focal nodular hyperplasia (FNH).

**Methods:** A retrospective analysis of the clinical data of patients undergoing US-guided percutaneous thermal ablation of FNH from November 2008 to August 2021 at five medical centers in China was conducted.

**Results:** A total of 53 patients were included (26 males and 27 females). The mean age was 35.1 ± 10.8 years. Sixty-five lesions (46 solitary cases and 7 cases with multiple lesions) were included, 70.8% (46/65) of which were located in the right liver lobe. The mean tumor length was 2.9 ± 1.5 cm. All patients successfully completed the ablation treatment. Immediate postoperative imaging showed that the primary technical success rate was 94.3% (50/53). Two patients underwent ablation 3 and 6 months after the primary ablation, and the secondary technical success rate was 100% (2/2). The incidence of complications was 3.8% (2/53). Imaging follow-up was conducted regularly after ablation, and no residual lesion enlargement or tumor recurrence was observed during the follow-up period. The technique efficacy rate was 98.1% (52/53).

**Conclusion:** US-guided percutaneous thermal ablation is a safe and effective treatment for FNH of the liver.

## Introduction

Focal nodular hyperplasia (FNH) is the second most common benign liver tumor in adults, with a prevalence of 0.3–3% in the general population (Oldhafer et al., 2020; Zhu et al., 2021). The exact mechanism of FNH is still unclear, but most scholars agree that nonneoplastic hyperplasia of hepatic parenchyma, which can be caused by anomalous aortic vascularization, secondary thrombosis, reactive hyperplasia after hepatocellular injury caused by vasculitis, or abnormal blood perfusion, may develop into FNH (Towbin et al., 2011; Franchi-Abella et al., 2013; Zarfati et al., 2020; Yao et al., 2021). Since FNH is usually asymptomatic and there have been no reports of malignant progression of this disease, conservative observation should be considered first. However, clinical treatment should be considered if the diagnosis is unclear, the patient has symptoms or the lesion becomes enlarged during follow-up (Perrakis et al., 2017; Jung et al., 2019).

Surgical resection and transarterial embolization (TAE) are the common treatment methods for FNH. Surgical resection has consistently been considered the preferred treatment, but it may cause greater damage and has a moderate incidence of complications and postoperative mortality (Virgilio and Cavallini, 2018), which may cause concerns for some patients. TAE can be applied to patients who are ineligible for resection and in cases where it is desirable to preserve the normal liver parenchyma. It is also commonly used to reduce the volume of lesions and control pain before surgery (Amesur et al., 2009; Arts et al., 2010; Virgilio and Cavallini, 2018), but TAE also has the risk of residual (Zhang et al., 2017) and increased radiation exposure.

Thermal ablation, a minimally invasive approach, has been widely applied in the treatment of small hepatocellular carcinoma and other solid tumors. Its advantage lies in its curative effect, minimal invasion and lighter economic burden. Theoretically, thermal ablation can also induce curative effects in FNH patients, but there have been few reports (Hedayati et al., 2010; Yao et al., 2021) about using ablation for treating FNH patients.

Therefore, this study aims to analyze the efficacy of US-guided thermal ablation for FNH by assessing clinical data of FNH patients from five medical centers.

## Materials and Methods

### Patients

**FIGURE 1:**
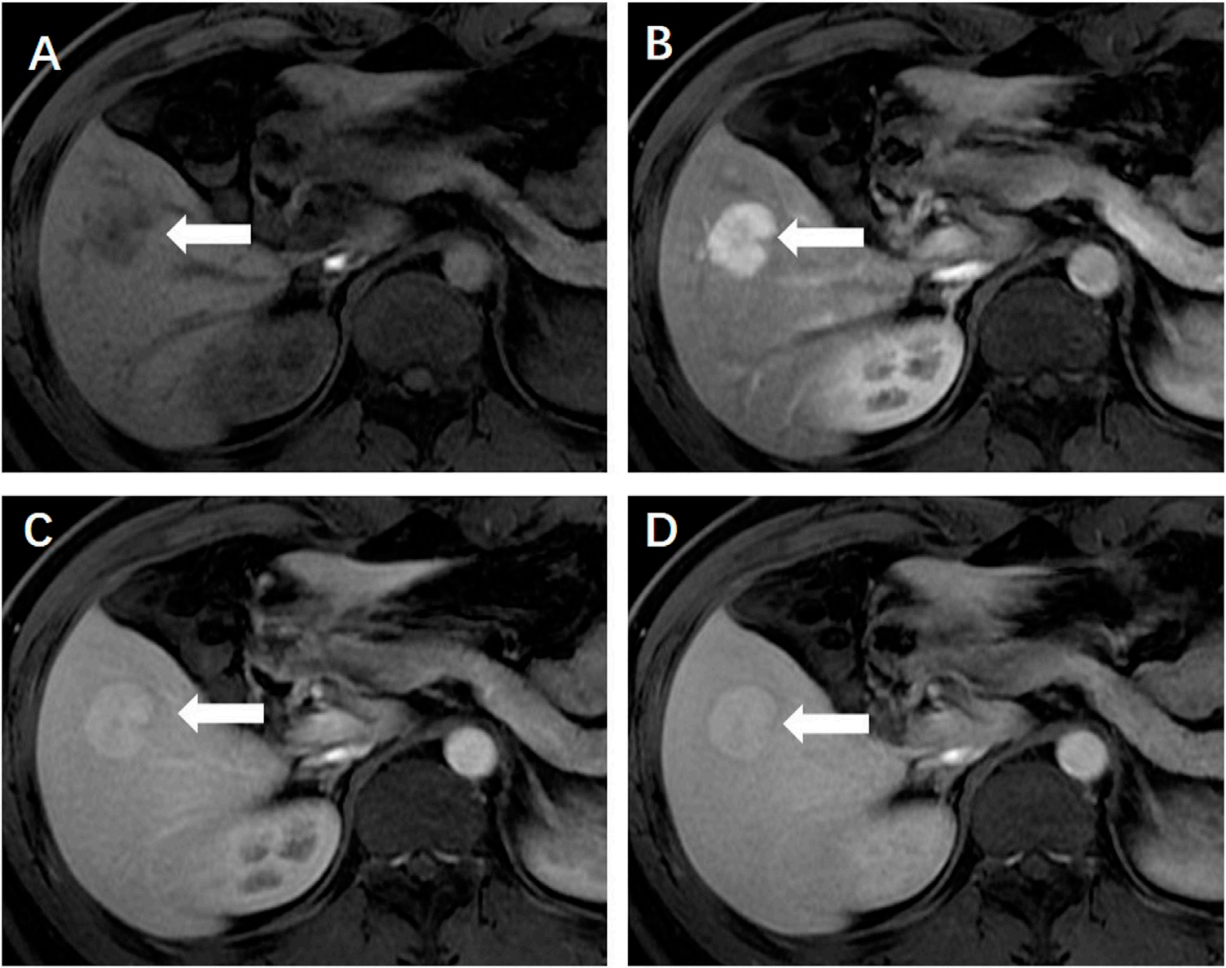
**(A)** Enhanced magnetic resonance imaging revealed a solid focal liver lesion (←) which showed hyperenhancement in the arterial phase **(B)** and isointensity in the portal venous **(C)** and delayed phase **(D)**.

**FIGURE 2:**
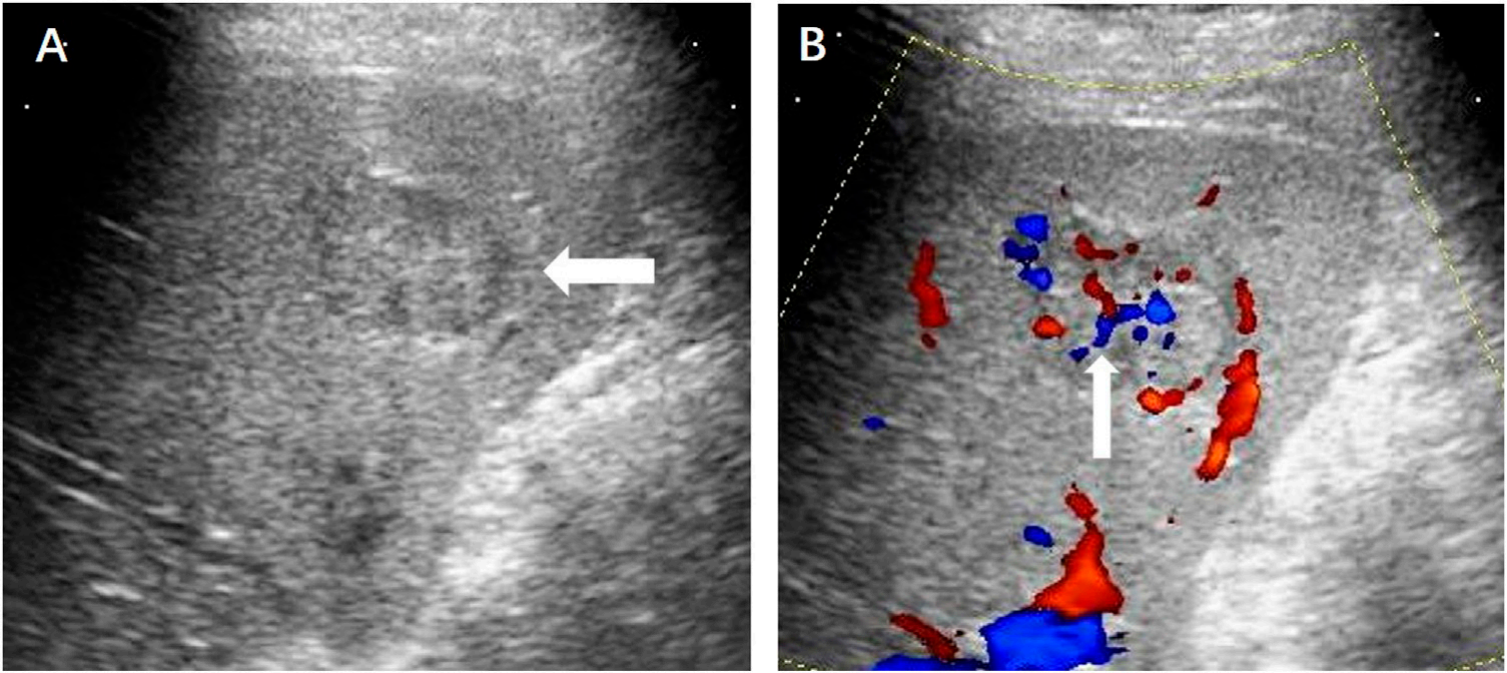
**(A)** Ultrasound revealed a hypoechoic hepatic lesion (←). **(B)** The lesion had a characteristic radiant blood flow (↑) shown by Color Doppler ultrasound.

**FIGURE 3:**
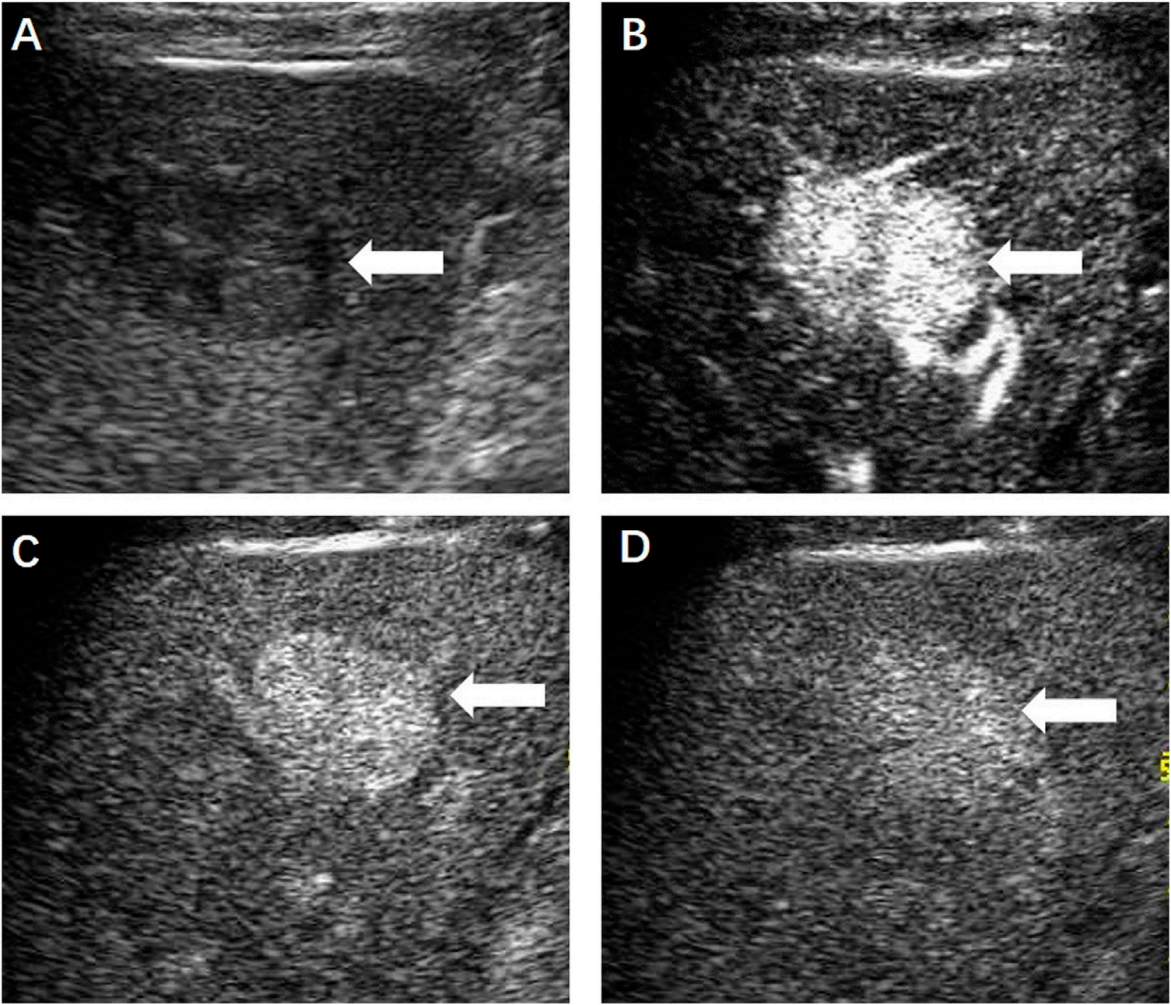
**(A)** Contrast-enhanced ultrasound displayed a hypoechoic hepatic lesion (←) which showed obvious hyperenhancement in the arterial phase **(B)** and slightly enhancement in the portal **(C)** and delayed phase **(D)**.

**FIGURE 4:**
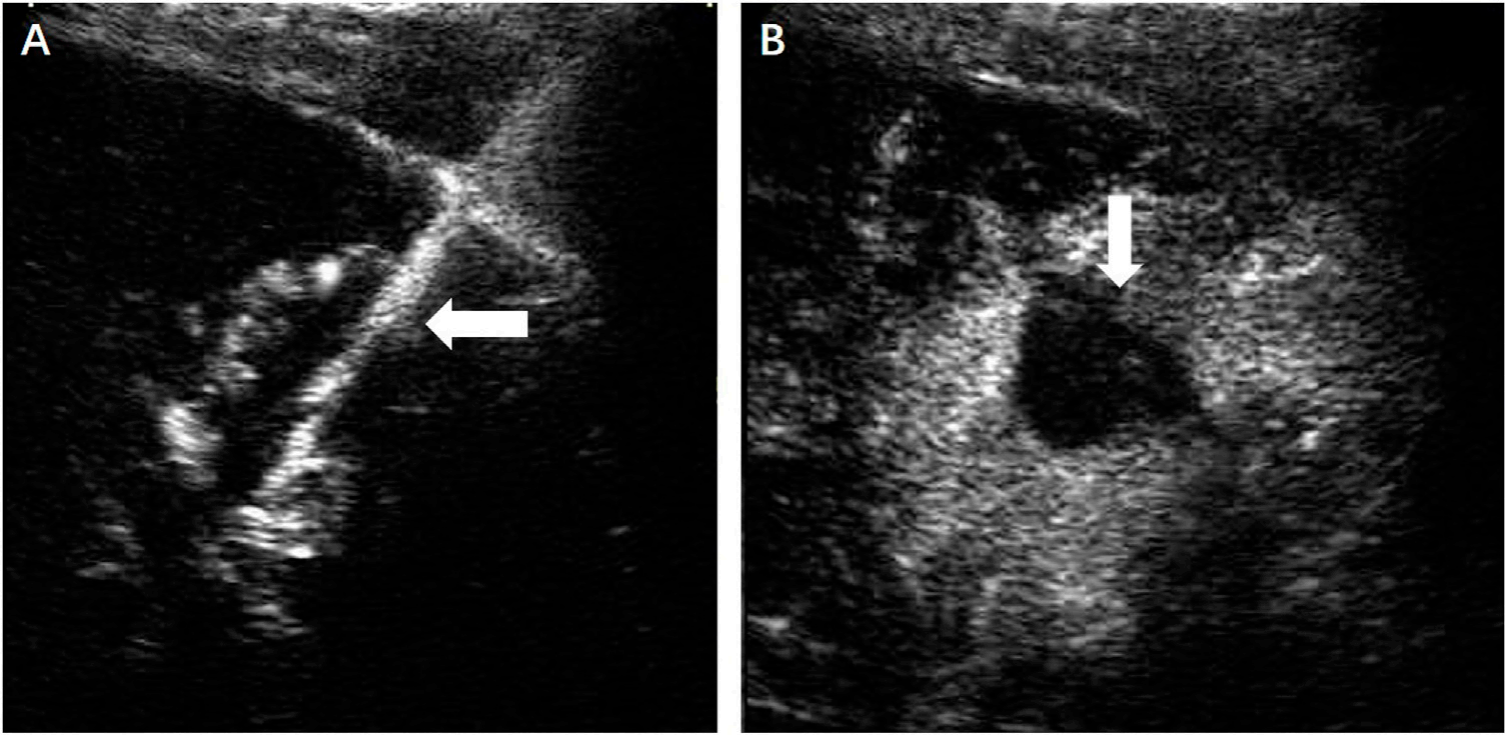
**(A)** Under the guidance of contrast-enhanced ultrasound, the microwave antenna (←) was inserted directly to the lesion. **(B)** No enhancement of the ablation zone (↓) could be seen in the arterial phase.

The study was approved by the Ethical Review Board of the Third Affiliated Hospital of Sun Yat-sen University. We performed a retrospective analysis of patient data using uniform data tables in five medical centers in China. The inclusion criteria for the study were as follows: 1) a diagnosis of FNH confirmed by pathological biopsy, or typical imaging characteristics (Figures 1–3) shown by contrast-enhanced ultrasound (CEUS) and hepatic magnetic resonance imaging (MRI); and 2) application of US-guided percutaneous thermal ablation (Figure 4A). The exclusion criteria were as follows: 1) application of thermal ablation combined with other treatments, such as surgical resection or TAE, on lesions diagnosed as FNH; and 2) lack of postoperative follow-up. The basic information included age, sex, and the number, location, and size of the lesions, and biopsy results, and information related to the ablation procedure was collected.

### Assessment of Therapeutic Efficacy and Follow-Up

**FIGURE 5:**
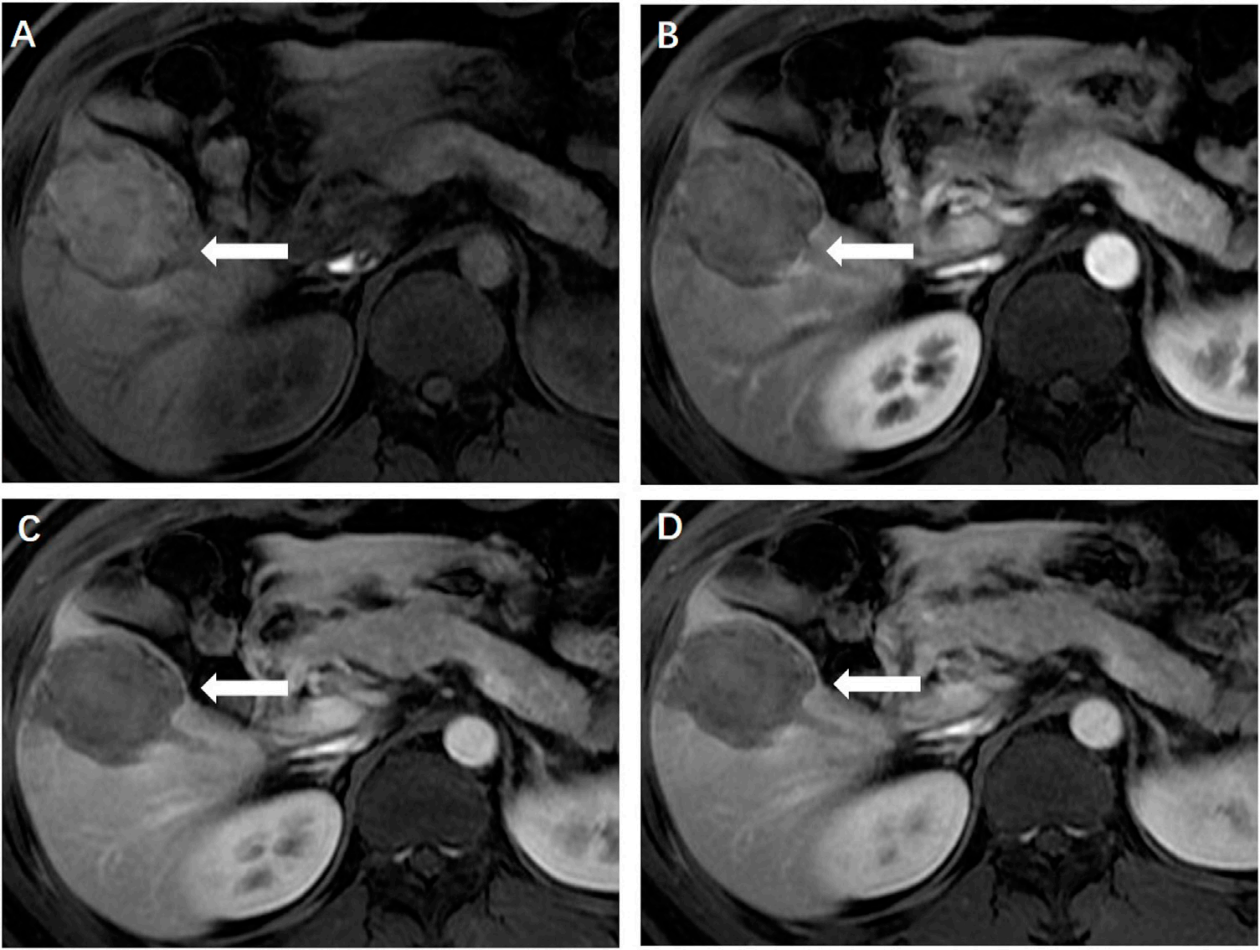
**(A)** Magnetic resonance imaging 1 month later showed the ablation zone was isointensity (←) and had no enhancement in the arterial **(B)**, portal venous **(C)** and delayed phase **(D)**, indicating completely ablation of the lesion.

The efficacy of thermal ablation was evaluated by US, computed tomography (CT) or MRI. Technical success was defined as the absence of blood supply in the ablation zone assessed by CEUS immediately after the treatment (Figure 4B) and the first postoperative day. After discharge, imaging follow-up was conducted regularly among all the patients until September 2021. Technique efficacy was defined based on a lack of enhancements of the primary lesion seen on enhanced-CT or MRI (Figure 5) at least 1 month after ablation.

## Results

### Patient Profiles

A total of 53 patients were enrolled between November 2008 and August 2021. The baseline characteristics of the patients are presented in Table 1.

**TABLE 1:** Baseline patient and lesion characteristics.

Variables	Numbers
patients	53
Age (years)	35.1 ± 10.8 (18–63)
Gender	
Male	26 (49.1%)
Female	27 (50.9%)
Lesions	
Solitary	46 (86.8%)
Multiple total	7 (13.2%)
Total	65
Tumor location	
Left	19 (29.2%)
Right	46 (70.8%)
Tumor size (cm)	2.9 ± 1.5 (0.6–6.6)
Biopsy	
Yes	39 (73.6%)
No	14 (26.4%)

Data are presented as the mean ± SD (range) or n (%).

### Operation and Outcomes

All the patients underwent US-guided percutaneous thermal ablation, including 31 patients who received microwave ablation (MWA), 20 patients who received radiofrequency ablation (RFA) and two patients who received RFA combined with MWA. Artificial ascites (15/53), one-lung ventilation (2/53) and laparoscopy (3/53) were used for assistance. All patients completed the ablation treatment successfully. Immediate postoperative CEUS showed that the ablation zone covered the initial lesions in 50 cases, which indicated that the primary technical success rate was 94.3% (50/53). Partial residual lesions were seen in three patients, two of whom underwent secondary ablation 3 and 6 months after the first procedure. The secondary technical success rate was 100% (2/2).

### Complications

The incidence of complications was 3.8% (2/53). One patient had intraoperative needle tract bleeding, which could be controlled by ablation. The other patient developed acute renal failure. After proper specialized treatment, the patient was discharged 1 month later. The rest of the patients did not experience severe complications. Some of the patients (24.5%, 13/53) had minor adverse reactions such as pain, slightly elevated aminotransferase levels and discomfort in the abdomen after ablation, which spontaneously resolved in a short time.

### Follow-Up

After ablation, all the patients were regularly followed up with imaging tests including US, CT or MRI. The residual part of the lesion in one patient showed no obvious changes on 1-month CEUS and 3-month MRI, so the patient did not undergo secondary ablation. The rest of the patients showed no evident residual lesions or tumor recurrence during the follow-up period. The technique efficacy rate was 98.1% (52/53). Furthermore, long-term complications related to ablation did not occur in any patient.

## Discussion

Thermal ablation, including RFA and MWA, is a major radical method for hepatocellular carcinoma and liver metastasis. A retrospective cohort study (Wang et al., 2016) of 221 patients analyzed the efficacy of MWA for liver cancer, with 90.95% of patients achieving initial complete ablation at the technical evaluation 1 month after surgery. You et al. used thermal ablation to treat 85 liver tumors, and the technical success rate was 100% (You et al., 2021). One instance of a residual lesion was found by CECT/CEMRI 1 month after surgery, and the technique efficacy rate was 98.8%. Technically successful ablation can provide 50–70% 5-year overall survival for very early and early HCC patients (Meloni et al., 2021). FNH is characterized as benign in nature but has abundant arterial blood supply, compared with liver cancer. Technically, thermal ablation can also be considered when clinical treatment is indicated in FNH patients. Hedayati et al. reported the first case of FNH treated with RFA in 2010 (Hedayati et al., 2010). The patient was a 21-year-old female with a history of progressive right upper abdominal pain. The size of the lesion was 2.2 cm and RFA was completed under the guidance of CT. Although a small nodular area of rim enhancement was noted on the follow-up CT 2 months later, the patient’s symptoms were significantly relieved. Overall, the treatment was considered successful. In our study, all patients were treated with US-guided percutaneous thermal ablation with a primary technical success rate of 93.9%, and two patients also achieved technical success after secondary ablation. Only one patient decided not to undergo secondary ablation. Most lesions were discovered accidentally, and only a few patients had obvious symptoms, which were significantly relieved after thermal ablation. During the follow-up period, there were no clear residual lesions or tumor recurrence in any patients except in one patient who did not achieve technical success after the first ablation. The overall technique efficacy rate was 98.1% (52/53). Our results suggest that, similar to the situation in liver cancer, thermal ablation for FNH can also achieve curative effects. In addition, our study is the first retrospective analysis of multicenter US-guided thermal ablation for FNH patients, and the results also provide a theoretical basis for prospective studies in the future.

For patients with unresectable lesions or who are ineligible for surgery, TAE is an alternative treatment. In 2013, Birn et al. reported a total of 17 lesions in 12 patients with FNH treated with TAE (Birn et al., 2013). The symptoms were completely relieved in seven patients and partially relieved in five patients after embolization. Only five of the 17 lesions were completely embolized based on a comparison with the lesion appearance on preoperative imaging. Virgilio et al. summarized 17 studies on the use of TAE in the treatment of FNH (Virgilio and Cavallini, 2018). A total of 128 patients received effective treatment, and each patient received TAE at least once. Local recurrence was found in only one of the treated patients during 54 months of follow-up. Although TAE is an effective treatment for FNH, there is a risk of increased radiation exposure in some specific patients, such as children. Our team reported a case of a 9-year-old girl who was diagnosed with FNH and treated by thermal ablation (Yao et al., 2021). The maximum diameter of the lesion was 2.9 cm, and it was treated by US-guided microwave ablation. Both the immediate postoperative CEUS and 1-month MRI after ablation showed that the lesion had been completely ablated. Similarly, in this study, 97.9% of the patients achieved complete remission of symptoms after one or two ablations and no recurrence of lesions was found during the current follow-up period. These findings indicated that thermal ablation, which is also a minimally invasive technique, can achieve therapeutic effects comparable to TAE and a lower residual lesion rate. Moreover, US-guided percutaneous thermal ablation has the advantage of no radiation exposure and may be more suitable for children or patients who want to reduce the risk of radiation exposure.

In terms of safety, complications of surgical resection mainly include intraoperative and postoperative bleeding, biliary fistula, intestinal obstruction, etc., with a mortality rate of approximately 2%. Although it is currently the preferred treatment for FNH, some scholars (Virgilio and Cavallini, 2018) believe that surgical resection-related complications and mortality are serious and unacceptable for the treatment of benign diseases. The common complications of thermal ablation for liver cancer mainly include subcapsular hematoma, abdominal skin burn and pleural effusion (Spiliotis et al., 2021). These complications may also occur during FNH treatment. However, in this study, only two patients had complications. One patient was found to have needle tract bleeding during the operation, and timely treatment with ablation stopped the bleeding. The other patient developed acute renal failure after surgery, which was relieved by specialized treatment. The rest of the patients did not develop severe complications. Some patients had postoperative adverse reactions, such as wound pain, mildly elevated aminotransferase levels, and discomfort in the upper abdomen, but these symptoms spontaneously resolved in a short time. Similar to thermal ablation, TAE is a relatively safe treatment. Postembolization syndrome, which is characterized by fever, loss of appetite, abdominal pain, low-grade fever, or nausea, is a common complication of TAE, but the symptoms are transient and self-limited (Virgilio and Cavallini, 2018). There is no literature comparing the safety of TAE and thermal ablation. The results of this study indicate that thermal ablation is a safe treatment with less trauma and a lower complication rate than surgical resection. Compared with that of TAE, the safety of thermal ablation is not significantly lower.

However, there are some limitations in this study. 1) Our study is a retrospective study, and only 53 cases were included. If thermal ablation is to be widely applied in the clinical treatment of FNH patients, more cases are needed to prove its effectiveness and safety. 2) This study only included patients treated with thermal ablation and failed to compare them with patients treated with surgical resection or TAE; as such, the results are not sufficiently convincing.

In general, US-guided percutaneous thermal ablation is an effective and safe treatment, and can be used as a radical treatment for FNH patients with appropriate treatment indications.

## Data Availability

The original contributions presented in the study are included in the article/Supplementary Material, further inquiries can be directed to the corresponding authors.
